# Enhancement of chemotherapeutic efficacy via non-canonical autophagy induced by Olea europaea in human model of lung adenocarcinoma cells (A549)

**DOI:** 10.3389/fcell.2026.1760977

**Published:** 2026-04-24

**Authors:** Maria del Pilar Romano, Rosita Di Palma, Matteo Ruzza, Francesco Albano, Pellegrino Mazzone, Rosalinda Sorrentino, Concetta Ambrosino, Geppino Falco, Mariarita Brancaccio

**Affiliations:** 1 Dipartimento di Biologia, Università degli Studi di Napoli Federico II, Napoli, Italy; 2 Istituto di Ricerche Genetiche Gaetano Salvatore Biogem Scarl, Ariano Irpino, Italy; 3 Dipartimento di Farmacia, Università di Salerno, Fisciano (SA), Italy

**Keywords:** chemotherapy, LC-3, lung cancer, natural compounds, non-canonical autophagy, Nrf-2, Olea europaea, ROS

## Abstract

**Introduction:**

Lung cancer (LC) remains a leading cause of cancer-related mortality worldwide, with poor prognosis and limited therapeutic efficacy due to drug resistance and off-target effects. Combining natural compounds with conventional chemotherapy is emerging as a promising strategy to enhance anticancer activity while reducing toxicity.

**Methods:**

We investigated the effects of an olive fruit extract from *Olea europaea* L. (OE) combined with 5-fluorouracil (5-FU) or cisplatin (CIS) on human lung adenocarcinoma A549 cells. Analyses included cell viability, autophagy markers, gene expression, ROS levels, and Nrf2 nuclear translocation.

**Results:**

Co-treatment with OE and chemotherapeutic agents significantly reduced cell viability and induced non-canonical autophagy, indicated by increased LC3BII and decreased Beclin-1 expression, without activating classical apoptotic markers. Gene expression analysis revealed downregulation of AGR2 and upregulation of GRP78, suggesting modulation of ER stress. ROS levels decreased, and Nrf2 nuclear translocation confirmed activation of the antioxidant response.

**Discussion:**

These findings indicate that OE potentiates the therapeutic effects of 5-FU and CIS by promoting alternative cell death pathways and modulating oxidative and ER stress responses. Our results support the potential use of *Olea europaea* as a safe and effective adjuvant in lung cancer treatment, providing a basis for further preclinical and clinical studies.

## Graphical Abstract

**Figure:**
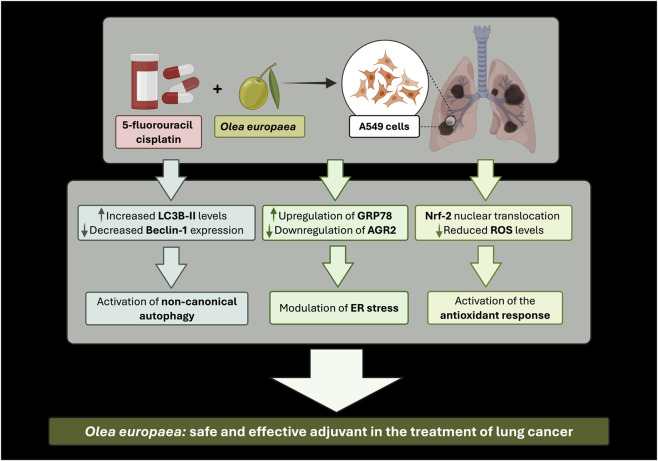

## 1 Introduction

Lung cancer, with almost 2.5 million new cases and over 1.8 million deaths, is the most commonly diagnosed cancer worldwide (Bray et al., 2024). Despite the growing interest in non-cytotoxic therapeutic agents, the standard treatment option for patients with advanced and unresectable non-small-cell lung cancer (NSCLC) remains chemotherapy (Andriani et al., 2006). Cisplatin (CIS) is considered the first-line chemotherapeutic agent for advanced NSCLC (Ashrafi et al., 2022), but its clinical usefulness is limited by the development of chemoresistance and cisplatin-associated toxicity (Bahuguna et al., 2017). On the other hand, 5-fluorouracil (5-FU), although not a standard treatment for NSCLC, has been evaluated in combination with other chemotherapeutic agents, including cisplatin, for its potential effects on lung cancer cells in advanced stages of the disease (Bareschino et al., 2011). This combination was initially hypothesized to enhance cancer cell cytotoxicity by targeting different DNA replication and repair mechanismlks; however, it has been associated with a significant increase in overall toxicity, raising concerns about its clinical utility for long-term or frontline treatment in NSCLC (Bayat et al., 2017). Despite these advancements, current therapies remain insufficient in improving long-term survival rates, highlighting the need to identify more effective and safer therapeutic strategies for NSCLC (Brancaccio et al., 2018).

Hence, alternative treatment approaches have been developed that focus on enhancing the anticancer potential of conventional chemotherapeutic drugs through combination therapies with natural compounds that can reduce or mitigate non-target toxicity (Brancaccio et al., 2019; Brancaccio et al., 2022). The World Health Organization estimates that over 80% of individuals in developing nations rely on traditional medicine for primary healthcare, and recent data indicate that more than 60% of cancer patients incorporate vitamins or herbal remedies into their treatment plans (Bray et al., 2024). As a result, phytochemicals have gained widespread attention as potential anticancer agents due to their ability to selectively target disease-specific pathways while causing fewer toxic effects and a reduced side-effect profile. Natural substances account for more than half of pharmaceutical drugs currently used in clinical settings, and many demonstrate notable anticancer properties (Browning et al., 2017). In recent years, research has increasingly focused on the potential of natural compounds to enhance the efficacy of conventional anticancer drugs, allowing lower dosages to achieve therapeutic outcomes while decreasing the likelihood of adverse effects associated with higher drug concentrations (Chung et al., 2012; Codogno et al., 2012; Foucquier et al., 2015). In particular, plant-derived compounds have been shown to target cancer cells by modulating programmed cell death pathways, including apoptosis and autophagy (Heckmann et al., 2017).

Among plant-derived natural products endowed with promising anticancer properties, *Olea europaea L*. has emerged as a particularly relevant source of bioactive compounds. Bioactive molecules derived from olive tree components, including leaves and fruits, have been proposed as adjuvants to conventional lung cancer therapies because they modulate oxidative stress, inflammation, and cell proliferation pathways. For example, combining olive leaf extract with cisplatin has been shown to decrease cell viability in lung cancer cells while protecting normal cells, potentially reducing chemotherapy-induced toxicity (Jaykaran, 2010; Klionsky et al., 2018). Oleuropein (OE), a major polyphenol found in olive leaves, has demonstrated anticancer effects across multiple cancer types, including lung cancer, by inhibiting cell proliferation, modulating apoptosis and autophagy, and regulating inflammatory pathways. Nevertheless, the molecular mechanisms underlying these effects—particularly in the context of combinatorial treatments and alternative autophagic responses—remain incompletely characterized. Notably, oleuropein has been reported to reduce cell viability in human lung cancer A549 cells by inducing oxidative stress and modulating nuclear factor kappa-light-chain-enhancer of activated B cells (NF-κB) signaling pathways (Klionsky et al., 2018).

Autophagy is a self-degradative cellular process in which cytoplasmic components are sequestered into autophagosomes, which are subsequently delivered to lysosomes for degradation and recycling (Kroemer et al., 2009). Beyond its primary role as a survival and quality-control mechanism, autophagy has also been recognized as a distinct mode of programmed cell death, known as type II cell death, distinct from apoptosis and necrosis (Kubben et al., 2016). Autophagy is regulated by a complex molecular network that includes canonical markers such as LC3B-I/II and Beclin-1. Importantly, autophagy may also proceed through non-canonical, Beclin-1–independent pathways, particularly under conditions of cellular stress. Reactive oxygen species (ROS), endoplasmic reticulum (ER) stress, and mitochondrial dysfunction are among the major triggers of autophagy activation (Kusuma et al., 2024).

In this context, key molecular biomarkers involved in ER stress–associated autophagy include anterior gradient 2 (AGR2) and glucose-regulated protein 78 (GRP78). Both proteins play critical roles in cellular stress adaptation and lung cancer progression and are closely linked to the ER stress response, which can ultimately lead to the activation of autophagic pathways. The interplay among AGR2, GRP78, and autophagy suggests that modulating these proteins may influence therapeutic sensitivity and treatment outcomes in lung cancer (Levine and Kroemer, 2008).

The present study investigates the adjuvant potential of Olea europaea–derived extracts, including olive leaf or fruit extracts rich in oleuropein, in combination with cisplatin and/or 5-fluorouracil in A549 human lung adenocarcinoma cells. The work focuses on elucidating the molecular mechanisms underlying the observed synergistic or additive effects of these combinatorial treatments, with particular attention to OE’s antioxidant properties, the modulation of oxidative and ER stress markers, and the activation of autophagy-related pathways. Special emphasis is placed on a Beclin-1–independent, non-canonical autophagic response observed *in vitro*, which may contribute to enhanced therapeutic efficacy while potentially limiting chemotherapy-associated toxicity.

## 2 Results

### 2.1 Plant-derived compound is not cytotoxic on human telomerase reverse transcriptase lung fibroblast (hTERT-LF)

**FIGURE 1:**
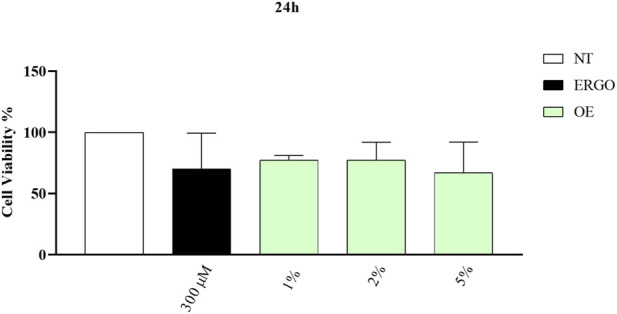
Plant natural compounds are not cytotoxic in hTERT-LF cells. Cells were treated with ERGO and OE for 24 h at the concentrations shown in the figure, and cell viability was measured by MTT assay. Data are expressed as mean ± SD (standard deviation), *n = 3*. The significance was determined by One-way ANOVA followed by Dunnett’s multiple comparisons test. No statistically significant differences were observed between the treatment groups and the control (p > 0.05).

To first evaluate any potential cytotoxicity of *Olea europaea L.* (OE) on healthy normal cells, human Telomerase Reverse Transcriptase Lung Fibroblast (hTERT-LF) were used (Figure 1). hTERT-LF cells were treated for 24 h with OE at 1%, 2%, and 5% concentrations. As a positive control, we used Ergothioneine (ERGO) at 300 μM. ERGO is a sulfur-containing histidine compound that has been shown to inhibit γ‐glutamyl transpeptidase (GGT) activity in human cancer cells at the concentration used. GGT inhibition plays a crucial role in cellular detoxification and oxidative stress regulation; therefore, ERGO is a relevant compound to use as a reference in this study, allowing us to compare the effects of the natural compound OE with those of a well-characterized molecule with antioxidant and metabolic regulatory properties. Additionally, the concentration used was chosen to ensure that ERGO exerts its metabolic effects without inducing cytotoxicity, providing a reliable control for assessing cell viability. Cell viability was then assessed by MTT assay, which evaluates the metabolic activity of living cells (Achi et al., 2022). The results showed that OE is not cytotoxic compared to untreated (NT) or the positive control (ERGO) (Figure 1). Although a slight decrease in cell viability was observed at all concentrations, OE did not induce any significant reduction compared to NT cells or cells treated with ERGO. Furthermore, cell viability was never greater than or equal to 50%, confirming that OE is not cytotoxic on hTERT-LF cells (Figure 1).

### 2.2 Plant-derived compound modulates viability in the A549 cell line

**FIGURE 2:**
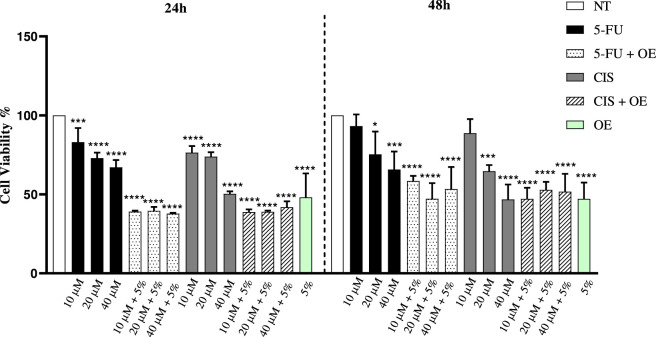
Effect of plant natural compounds, 5-Fluorouracil and Cisplatin, on the viability of A549 cells. Cells were treated for 24 and 48 h with OE, 5-FU, CIS, and the chemotherapeutic agents combined with OE at the concentrations shown in the figure, and cell viability was measured by an MTT assay. Data are expressed as mean ± SD (standard deviation), *n* = 3. The significance was determined by One-way ANOVA followed by Dunnett’s multiple comparisons test * (p *<* 0.05), *** (p *<* 0.001), and **** (p *<* 0.0001) represent significance compared to not treated (NT).

To investigate if plant-derived compound OE could inhibit the viability of A549 cells, the cells were treated with OE at a concentration of 5%, 5-FU, and CIS (negative controls) at concentrations ranging from 10 to 40 μM. The 5% concentration of OE was selected as the highest non-cytotoxic dose, as determined by MTT assay on healthy human fibroblasts, to maximize the bioactive potential of the extract while ensuring cellular safety. This concentration also allowed for optimized combinatorial analysis, enabling the assessment of potential synergistic or additive effects between OE and chemotherapeutic agents; on the other hand the selected concentrations of 5-FU and CIS were based on previous studies (Lee and Wu, 2012; Liang et al., 1999; Liu et al., 2017). Additionally, to test the potential synergistic effect of OE with 5-FU and CIS, cells were co-treated with 5% OE and either 5-FU and/or CIS (10–40 µM) for 24 and 48 h (Figure 2). Subsequently, cell viability was assessed by MTT assay (Laurino et al., 2023). At 24 h, a reduction in cell viability was already observed; in particular, OE alone showed a modest inhibitory effect, whereas the combination treatments showed a slight reduction in viability compared with 5-FU and CIS alone. No further decrease in viability was observed at 48 h. In fact, the viability values at 48 h were slightly higher than those at 24 h, suggesting that the treatment’s cytotoxic effect is more efficient at 24 h. Moreover, the data indicate an enhanced cytotoxic effect when OE is combined with conventional chemotherapeutic agents (Figure 2).

In addition, to assess the interaction between 5-FU+OE 5% and CIS+OE 5% on cell viability, the Bliss Independence model was applied (Livak and Schmittgen, 2001). This model estimates the expected combined effect assuming that the two agents act independently, allowing comparison between the predicted additive effect and the experimentally observed response. Raw cell viability values (%) for single treatments and their combinations, used for Bliss calculations, are reported in Supplementary S1.

The Bliss model was selected because it is particularly suitable for evaluating drug combinations with distinct, non-overlapping mechanisms of action (Bareschino et al., 2011). Under these conditions, Bliss Independence provides a biologically appropriate framework to assess whether the combined effects deviate from simple additivity.

Following that, the Bliss analysis revealed that the reduction in cell viability observed for the combined treatments was synergistic (E_AB_ > E_Bliss_) (see Table 1), supporting a functional interaction between OE and conventional chemotherapeutic agents. In particular, within 24 h of treatment, the experimental antiproliferative effect (E_AB_) of OE in combination with 5-FU or CIS exceeded the effect predicted by the Bliss Independence model (E_Bliss_), indicating a positive interaction compared with single treatments. Based on these results, subsequent molecular analyses were performed on cells treated for 24 h with the combination of the natural compound and the chemotherapeutic agents.

**TABLE 1:** Calculation of the synergic effect of 5-FU+OE 5% and CIS+OE 5% in A549 cells treated for 24 h by the Bliss method (Livak and Schmittgen, 2001).

Treatments	E_*AB*_	E_*Bliss*_
5-FU 10 μM + OE 5%	0.61	0.14
5-FU 20 μM + OE 5%	0.60	0.14
5-FU 40 μM + OE 5%	0.62	0.14
CIS 10 μm + OE 5%	0.61	0.14
CIS 20 μM + OE 5%	0.61	0.12
CIS 40 μM + OE 5%	0.58	0.12

### 2.3 Impact of plant-derived compound on apoptosis in the A549 cell line

**FIGURE 3:**
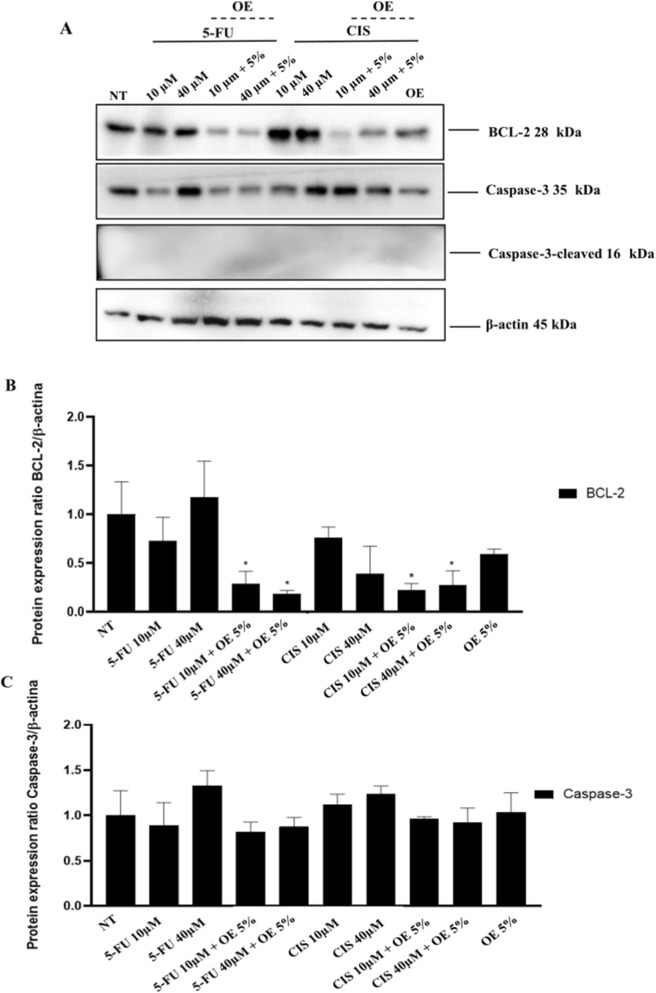
Plant-derived compounds may shift the balance away from classical apoptotic pathways in A549 cells. **(A)** Western blot analysis of Bcl-2 and Caspase-3 and Cleaved Caspase-3 expression in A549 cells treated for 24 h with indicated doses of OE, 5-FU, CIS, and treatments in combination. Bands are representative of one out of three separate experiments performed; **(B)** Densitometric analysis: Bcl-2 data were normalized to β-actin. Data are expressed as mean ± SD (standard deviation), *n* = 3. The significance was determined by One-way ANOVA followed by Dunnett’s multiple comparisons test. * (p < 0.05) represents significance compared to the not treated (NT) group. **(C)** Densitometric analysis: Caspase-3 data were normalized to β-actin. Data are expressed as mean ± SD (standard deviation), *n* = 3. The significance was determined by One-way ANOVA followed by Dunnett’s multiple comparisons test; no statistically significant differences were observed between the treatment groups and the control (p > 0.05).

To determine whether A549 cells treated with 5-FU, CIS, OE, and the co-treatment could lead to apoptotic cell death, apoptotic-specific markers (Caspase-3, Cleaved Caspase-3, and Bcl-2) were analyzed by immunoblotting (Figure 3A). Monitoring Bcl-2 revealed that the levels of this anti-apoptotic protein in cells treated with either the chemotherapeutic agent alone or OE alone were comparable to those in untreated cells and showed no statistically significant changes (Figure 3B). On the other hand, cells co-treated with the chemotherapeutic agent and OE exhibited a substantial decrease in Bcl-2 expression (Figure 3B). Although a significant reduction in Bcl-2 levels would typically induce apoptosis, the absence of Cleaved Caspase-3 (Figure 5A) and the presence of Caspase-3 in its inactive form (Figure 3C) suggest that the reduction in Bcl-2 does not trigger a complete apoptotic cascade. Instead, it may promote autophagy by releasing Beclin-1, which is usually sequestered by Bcl-2 (Lockshin and Zakeri, 2004). The boundary between apoptosis and autophagic cell death has not been definitively established, and there is an overlap between these two types of cell death; autophagy can end with apoptosis, and apoptosis can begin with autophagy (Mariño et al., 2014). This suggests that the co-treatment may shift the balance away from classical apoptotic pathways, potentially activating a canonical or non-canonical form of autophagic cell death.

### 2.4 Plant-derived compound activates autophagic processes in the A549 cell line

**FIGURE 4:**
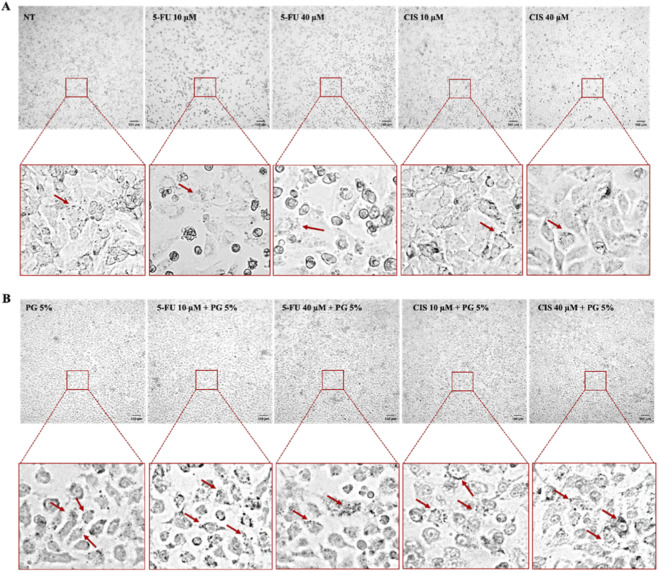
Morphological changes in A549 cells after treatments. **(A)** A549 cells not treated (NT), treated with 5-FU and CIS 10 and 40 µM showed a discrete number of vacuoles (indicated with red arrow). **(B)** A549 cells treated with 5% OE and co-treated with 5% OE and 10 and 40 µM of 5-FU and CIS showed an increase in vacuoles (indicated with red arrow).

Morphological changes were observed after treating A549 cells with 5% OE, 5-FU, or CIS at concentrations ranging from 10 to 40 μM, as well as with the co-treatments, for 24 h, as shown in Figure 4, using the Apexview APX100 (Olympus, Japan). The increase in vacuoles within A549 cells treated with OE and co-treatments suggested an autophagic process activation (Figure 4B).

**FIGURE 5:**
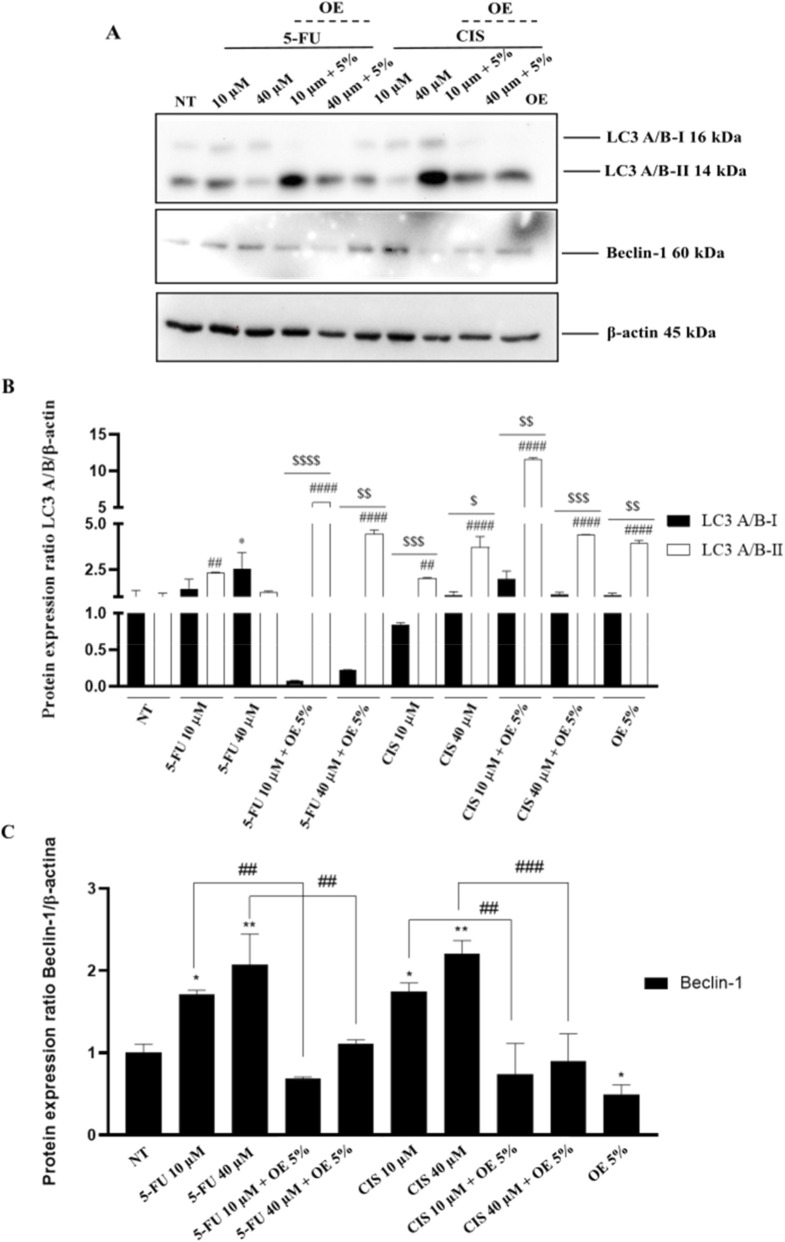
Plant-derived compounds induce a non-canonical autophagy pathway in A549 cells. **(A)** Western blot analysis of LC3A/B-I and LC3A/B-II expression in A549 cells treated for 24 h with indicated doses of OE, 5-FU, CIS, and combined treatments. Bands represent one out of three separate experiments performed. **(B)** Densitometric analysis. LC3A/B data were normalized to β-actin. Data are expressed as mean ± SD (standard deviation), *n* = 3. The significance was determined by One-way ANOVA followed by Dunnett’s multiple comparisons test * (p < 0.05) represents significance LC3A/B-I compared to not treated (NT); ## (p < 0.01), ### (p < 0.001) and #### (p < 0.0001) represents significance LC3A/B-II compared to not treated (NT); finally, the significance was determined by the Student’s t-test $ (p < 0.05), $$ (p < 0.01), $$$ (p < 0.001) and $$$$(p < 0.0001), represent significance of LC3A/B-I compared to LC3A/B-II. **(C)** Densitometric analysis. Beclin-1 data were normalized to β-actin. Data are expressed as mean ± SD (standard deviation), *n* = 3. The significance was determined by One-way ANOVA followed by Dunnett’s multiple comparisons test * (p < 0.05) represents significance compared to not treated (NT); the significance was determined by the Student’s t-test $$ (p < 0.01) represents significance compared to 5-FU and CIS 10 μM, ## (p < 0.01) and ### (p < 0.001) represents significance compared to 5-FU and CIS 40 µM.

One approach to monitor autophagy is to detect LC3A/B conversion (LC3A/B-I to LC3A/B-II) by immunoblotting analysis, as the amount of LC3A/B-II is strongly associated with the number of autophagosomes and is considered the hallmark of mammalian autophagy (Milito et al., 2019). To verify this hypothesis, autophagy-specific markers (LC3A/B, Beclin-1) were analyzed by immunoblot analysis to examine the autophagic levels and flux. As shown in Figures 5B,C an upregulation of Beclin-1 and an increase in LC3A/B-I to LC3A/B-II conversion were observed in A549 cells treated with 10 and 40 µM of 5-FU and CIS, suggesting the activation of canonical autophagy. On the other hand, in cells co-treated with 5-FU or CIS and OE, there is a significant decrease in Beclin-1 along with a considerable increase in LC3A/B-II, suggesting that autophagy occurs in a Beclin-1-independent manner, indicative of a non-canonical form of autophagic cell death. These results indicate that OE-induced autophagy is not dependent on the Beclin-1 canonical pathway.

To further strengthen the hypothesis of a non-canonical autophagic pathway, ATG7 gene silencing was performed, as ATG7 is a key protein in the LC3 conjugation system of the canonical autophagy pathway. Transfection efficiency was first verified by immunoblotting, which showed a marked reduction in ATG7 protein levels in siATG7-transfected cells compared to the Scramble control, confirming successful knockdown (see Supplementary Figure S1).

Subsequently, ATG7-silenced A549 cells were treated with CIS, 5-FU, OE, and their respective combinations. Analysis of LC3A/B levels revealed that LC3-I to LC3-II conversion was still detectable in non-treated (NT) cells, Scramble control cells, and in cells treated with CIS and 5-FU at both concentrations. This finding suggests that, under these conditions, autophagic activation may occur independently of ATG7 or that residual ATG7 activity is sufficient to sustain basal LC3 processing. In contrast, in co-treated cells (CIS+OE and 5-FU+OE) as well as in cells treated with OE alone, no LC3-II activation was observed under ATG7-silenced conditions (see Supplementary Figure S1).

Regarding Beclin-1, no detectable expression was observed in any of the analyzed conditions, consistent with previous findings (see Supplementary Figure S1).

Overall, these results indicate that while single chemotherapeutic treatments appear to retain an ATG7-dependent autophagic component, the combination with OE fails to sustain LC3 activation in the absence of ATG7. The concomitant lack of Beclin-1 further supports the hypothesis that the co-treatment may modulate an alternative autophagic mechanism distinct from the canonical Beclin-1-dependent pathway, highlighting a complex regulatory framework that does not fully overlap with classical autophagic pathways.

In addition, to verify autophagy activation, we treated cells with 3-methyladenine (3-MA), a potent inhibitor of autophagy.

**FIGURE 6:**
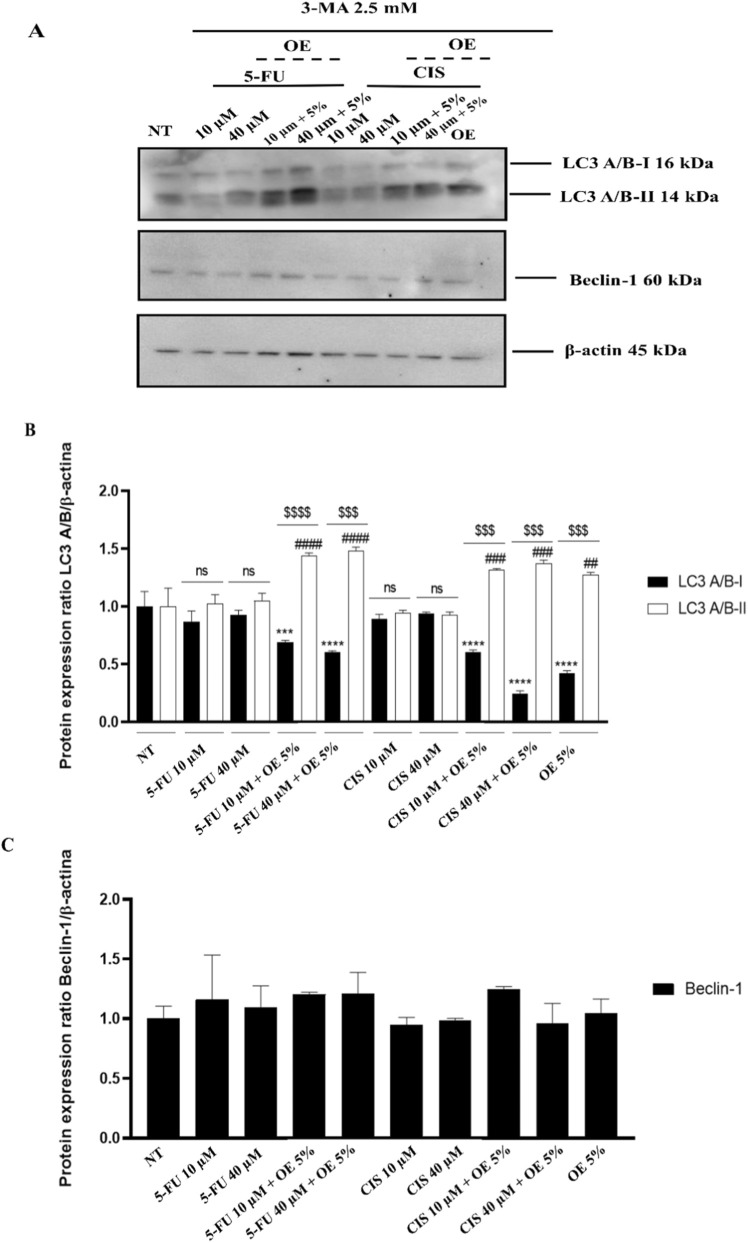
Activation of a non-canonical autophagy pathway in A549 cells after 3-MA treatment. **(A)** Representative Western blot of LC3A/B-I, LC3A/B-II, and Beclin-1 expression in A549 cells treated for 24 h with indicated doses of OE, 5-FU, CIS, and combined treatments, and after inhibiting autophagy by 3-MA. **(B)** Densitometric analysis. LC3A/B data were normalized to β-actin. Data are expressed as mean ± SD (standard deviation), *n* = 3. The significance was determined by One-way ANOVA followed by Dunnett’s multiple comparisons test *** (p < 0.001),****(p < 0.0001) represents significance LC3A/B-I compared to not treated (NT); ## (p < 0.01), ### (p < 0.001) and #### (p < 0.0001) represents significance LC3A/B-II compared to not treated (NT); finally, the significance was determined by the Student’s t-test $$$ (p < 0.001) and $$$$(p < 0.0001), represent significance of LC3A/B-I compared to LC3A/B-II. **(C)** Densitometric analysis. Beclin-1 data were normalized to β-actin. Data are expressed as mean ± SD (standard deviation), *n* = 3. The significance was determined by One-way ANOVA followed by Dunnett’s multiple comparisons test.

In particular, we first treated the A549 cells with the indicated concentrations of OE, 5-FU, CIS, and combined treatments for 24 h (see Figure 6), and subsequently treated them with 3-MA at 2.5 mM for 4 h, as reported in Liu et al. (Nooreldeen and Bach, 2021).

Following this approach, we observe that the combined treatment induces accumulation of LC3A/B-II compared to not treated (NT) and single treatments (Figure 6). We do not observe any significant modifications for Beclin-1 (Figure 6). These data confirm the activation of the alternative autophagy process. Given the known off-target effects of 3-MA, these findings were interpreted cautiously and considered supportive—but not definitive—evidence for alternative autophagic pathway activation (Pan et al., 2013).

### 2.5 Synergistic effects of *Olea europaea* and chemotherapy on Nrf2 activation and ROS reduction in A549 cells

**FIGURE 7:**
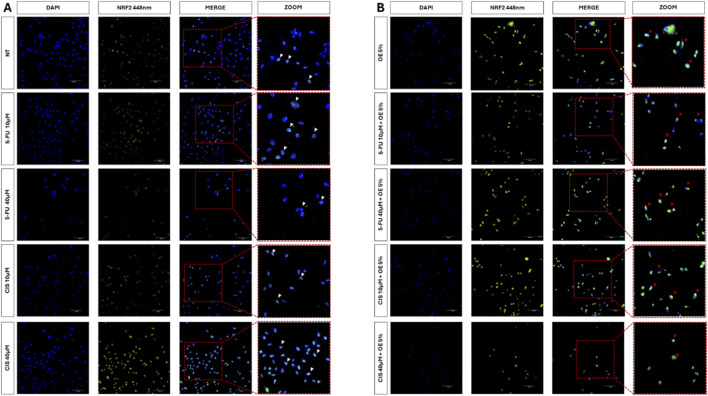
Activation of Nrf2. Immunofluorescence on A549 cells, evaluating the translocation of Nrf2 from the cytoplasm to the nucleus. In **(A)**, Cells were treated for 24 h with OE, 5-FU, and CIS at the concentrations shown in the figure; the white triangles represent the Nrf2 signal (point-like). In **(B)** Cells were treated for 24 h with chemotherapeutic agents combined with OE at the concentrations shown in the figure; red triangles, Nrf2 signal (accumulation). Images were taken using Evident FV4000 confocal microscope using a 20X objective. Scale bar 100 μm.

To clarify the behaviour of OE alone and in combination with 5-FU and CIS, we performed immunofluorescence on NF-E2-related factor-2 (Nrf-2), considering that Nrf-2 represents one of the master transcriptional regulators of the antioxidant and anti-inflammatory responses (see Figure 7).

In both Panel A and Panel B, we observe nuclear localization of Nrf-2; however, the signal in Panel B is stronger than in Panel A (see Figure 7).

**FIGURE 8:**
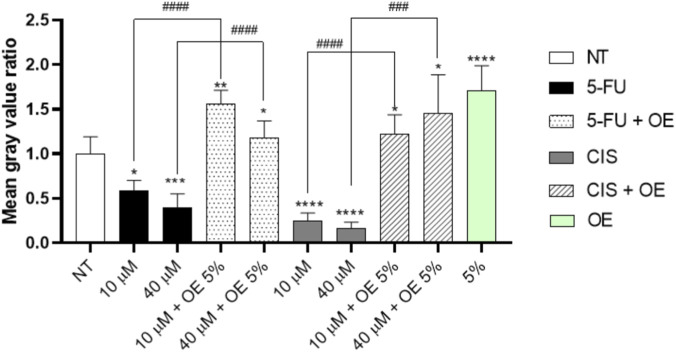
Fluorescence intensity expressed as mean intensity gray ratio (Y-axis) for each experimental condition. Data are expressed as mean ± SD (standard deviation), *n* = 3. The significance was determined by One-way ANOVA followed by Dunnett’s multiple comparisons test. ^*^ (p < 0.05), ^**^ (p < 0.01), ^***^ (p < 0.001) and, **** (p < 0.0001) represents significance compared to the not treated (NT) group. Student’s t-test comparing combination treatments to 5-FLU or CIS, ^#^ (p < 0.05), ^##^ (p < 0.01), ^###^ (p < 0.001), and ^####^ (p < 0.0001).

Following these observations by ImageJ, we analysed the intensity of the signal as reported in Figure 8.

In fact, we observed a significant increase in fluorescence in combo treatment both with 5-FLU and CIS (see Figure 8). At the same time, cells treated only with traditional chemotherapy have a decrease in fluorescence in comparison to NT (Figure 8).

On the other hand, to verify if A549 cells treated with 5-FU, CIS, OE, and the co-treatment could lead to ROS modulation following Nrf-2 activation, we performed the diclorofluorescein (DCF) assay.

**FIGURE 9:**
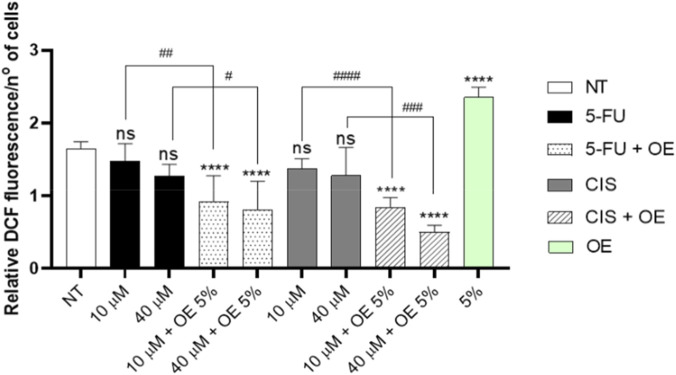
OE promotes antioxidant effects in combination with traditional chemotherapy in A549 cells. Cells were treated for 24 h with OE, 5-FU, CIS, and the chemotherapeutic agents combined with OE at the concentrations shown in the figure, and ROS production was measured by DCF assay. Data are expressed as mean ± SD (standard deviation), *n* = 3. The significance was determined by One-way ANOVA followed by Dunnett’s multiple comparisons test. **** (p < 0.0001) represents significance compared to the not treated (NT) group. Student’s t-test comparing combination treatments to 5-FLU or CIS, ^#^ (p < 0.05), ^##^ (p < 0.01), ^###^ (p < 0.001) and ^####^ (p < 0.0001).

We can observe a reduction in ROS in samples co-treated with 5-FU + OE and CIS + OE in comparison to NT (Figure 9); contemporary, in the cells treated only with OE, we can note an increase in ROS production in comparison to NT (Figure 9).

Probably, OE has shown a different role, and that depends on the context in which it works (Ruzzolini et al., 2018).

In fact, when used as a single treatment, OE may act as a pro-oxidant, inducing lethal oxidative stress in cancer cells. In contrast, in combination with 5-FU or CIS, OE appears to act as an antioxidant, reducing the excess ROS induced by chemotherapeutic agents and modulating the cellular stress response.

This apparent dual behavior suggests a context-dependent redox activity of OE, acting as a pro-oxidant when administered alone and as an antioxidant in the presence of chemotherapy-induced oxidative stress**.** Indeed, Nrf2 activation was detected in A549 cells following co-treatment with OE and either 5-FU or CIS and was associated with a significant reduction in intracellular ROS levels, indicating an enhanced antioxidant response. Mechanistic interpretations are therefore presented cautiously, distinguishing direct experimental observations from potential signaling interactions, while supporting OE’s modulatory role in oxidative stress responses during combined treatment.

### 2.6 Plant-derived compound affects gene expression of AGR2 and GRP78 in the A549 cell line

**FIGURE 10:**
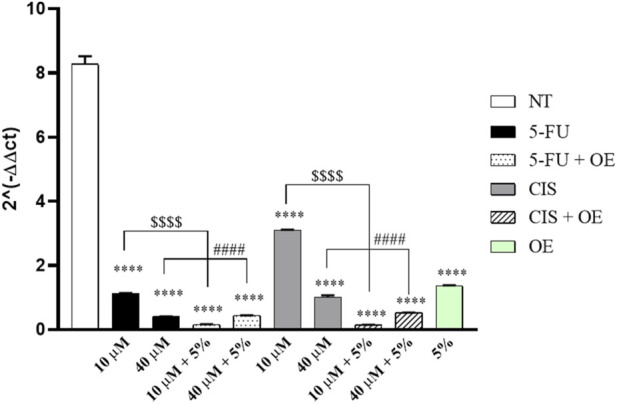
Gene expression of AGR2 in the A549 cell line. Data are expressed as mean ± SD (standard deviation), *n* = 3. The significance was determined by One-way ANOVA followed by Dunnett’s multiple comparisons test **** (p < 0.0001) represents significance compared to not treated (NT). The significance was determined by Student’s t-test; $$$$ (p < 0.0001) indicates significance compared to 5-FU and CIS 10 μM, and #### (p < 0.0001) indicates significance compared to 5-FU and CIS 40 µM.

To evaluate the modulation of gene expression of specific lung cancer biomarkers: AGR2 and GRP78, RT-qPCR analysis was carried out after treating A549 cells with 10 and 40 μM of 5-FU and CIS, 5% of OE and co-treatments of 5% OE with either 5-FU and CIS (10–40 µM) for 24 h. A significant decrease in AGR2 gene expression was observed in cells treated with 5-FU, CIS, OE alone, and in cells co-treated with chemotherapeutic agents and 5% OE compared to NT (Figure 10).

Furthermore, the co-treatments of 5-FU and CIS (10–40 µM) significantly reduced AGR2 gene expression compared to the treatment with chemotherapeutic agents alone.

**FIGURE 11:**
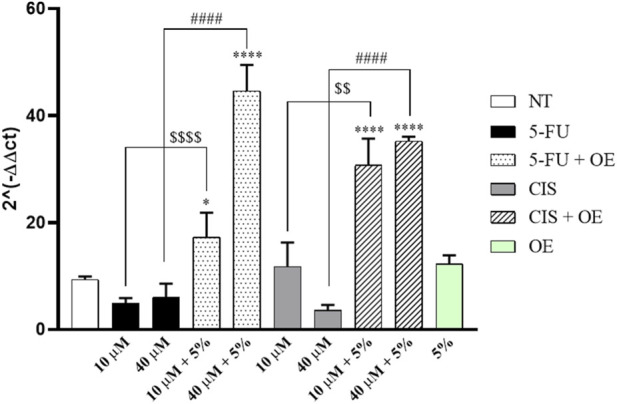
Gene expression of GRP78 in the A549 cell line. Data are expressed as mean ± SD (standard deviation), *n* = 3. The significance was determined by One-way ANOVA followed by Dunnett’s multiple comparisons test * (p < 0.05) and **** (p < 0.0001) represents significance compared to not treated (NT). The significance was determined by the Student’s t-test, $$$$ (p < 0.0001) and $$ (p < 0.01) represent significance compared to 5-FU and CIS 10 μM, #### (p < 0.0001) represents significance compared to 5-FU and CIS 40 µM.

On the other hand, gene expression analysis for GRP78 reveals its upregulation in cells co-treated with 5% OE and either 5-FU or CIS (10–40 µM) for 24 h (Figure 11).

## 3 Discussion

Autophagy plays a dual role in cancer, functioning both as a survival mechanism and as a pathway for cell death. Its complexity and context-dependent effects have hindered the development of targeted therapies (Liu et al., 2017). Canonical autophagy is typically dependent on Beclin-1, which interacts with Bcl-2 family proteins to regulate autophagosome formation. Under basal conditions, Bcl-2 binds Beclin-1 and represses autophagy; conversely, Bcl-2 inhibition or downregulation allows Beclin-1 to initiate autophagy, promoting LC3-II accumulation and autophagosome formation (Nooreldeena and Bach, 2021; Pan et al., 2013). However, autophagy can also proceed through non-canonical pathways that are independent of Beclin-1 or that diverge from the classical regulatory axis, although these mechanisms remain incompletely characterized (Kubben et al., 2016).

In this study, we investigated the effects of combining *Olea europaea* (OE) extracts with the chemotherapeutic agents 5-fluorouracil (5-FU) and cisplatin (CIS) in A549 human lung adenocarcinoma cells, focusing on autophagy, oxidative stress, and ER stress responses. Cytotoxicity assays demonstrated that OE is non-toxic to normal hTERT-LF cells, confirming its safety profile, while significantly enhancing the reduction of cancer cell viability when co-administered with 5-FU or CIS.

Immunoblot analysis revealed that Bcl-2 expression was significantly decreased in co-treated A549 cells, whereas single treatments (OE, 5-FU, CIS) did not significantly alter Bcl-2 levels compared to controls. Although Bcl-2 downregulation would typically favor apoptosis, Cleaved Caspase-3 was not detected, and Caspase-3 remained in its inactive form. These findings indicate that classical apoptosis was not the predominant mechanism of cell death despite the reduction in Bcl-2 expression (Scherz-Shouval and Elazar, 2007). Instead, Bcl-2 downregulation may have contributed to the release of autophagy-related signaling components.

Morphological analysis showed a marked increase in cytoplasmic vacuolization in co-treated cells, accompanied by significant LC3A/B-II accumulation, indicative of autophagosome formation. Notably, Beclin-1 expression was reduced in co-treated cells compared to single-agent treatments. These findings suggest that the autophagic response induced by the combination therapy does not fully rely on the canonical Beclin-1-dependent pathway (Segawa et al., 2000).

Importantly, ATG7 gene silencing provided further mechanistic insight. Efficient ATG7 knockdown was confirmed by immunoblotting. Under ATG7-silenced conditions, LC3-II accumulation persisted in non-treated cells, Scramble controls, and in cells treated with CIS or 5-FU alone, whereas LC3 activation was abolished in OE-treated and co-treated cells. In parallel, Beclin-1 remained undetectable across conditions. These results indicate that, while single chemotherapeutic agents may retain an autophagic component even after ATG7 reduction—possibly due to residual activity or compensatory mechanisms—the autophagic response triggered by OE-containing combinations is dependent on ATG7 but occurs independently of Beclin-1. Therefore, the data support the activation of a non-canonical autophagic process characterized by divergence from the classical Beclin-1 regulatory axis rather than a fully ATG7-independent alternative pathway.

Consistent with this interpretation, 3-MA treatment did not completely abolish LC3-II accumulation in co-treated cells. Considering the known off-target effects of 3-MA, these results were interpreted cautiously but further support the involvement of an alternative regulatory mechanism (Liu et al., 2017).

At the transcriptional level, RT-qPCR analysis showed that OE co-treatment downregulated AGR2, a gene frequently overexpressed in lung cancer and associated with poor prognosis. AGR2 reduction was observed both with chemotherapeutic agents alone and in combination with OE, suggesting a contribution of ER stress modulation to the antitumor response (Jaykaran, 2010).

Additionally, GRP78, a master regulator of ER stress and chaperone protein, was significantly upregulated in co-treated cells. GRP78 activation has been associated with autophagy induction and modulation of cancer cell migration, and in some contexts is considered cytoprotective. In our model, its induction may reflect an adaptive response to proteotoxic stress or participate in the regulation of autophagic signaling during combination treatment.

Furthermore, co-treatment with OE reduced intracellular ROS levels compared to chemotherapy alone. Given the well-established antioxidant properties of *Olea europaea*, this finding suggests that OE modulates the oxidative microenvironment induced by chemotherapeutic stress, potentially influencing redox-sensitive signaling pathways linked to autophagy regulation (Kubben et al., 2016).

Taken together, our data demonstrate that the combination of *Olea europaea* extracts with conventional chemotherapy enhances cytotoxic effects in A549 cells through modulation of a Beclin-1–independent but ATG7-dependent autophagic pathway, along with coordinated regulation of oxidative and ER stress responses. The decrease in Bcl-2 without activation of apoptotic markers, coupled with LC3-II accumulation and Beclin-1 suppression, supports a shift away from classical apoptosis toward a regulated autophagic cell death mechanism (Segawa et al., 2000).

These findings contribute to the growing body of evidence suggesting that natural compounds such as *Olea europaea* may function as therapeutic adjuvants, potentially improving chemotherapeutic efficacy through modulation of complex intracellular pathways—including autophagy, oxidative stress, and ER signaling—central to cancer cell fate determinat

## 4 Conclusion

This study provides preliminary evidence that *Olea europaea* (OE) extract enhances the cytotoxic effects of cisplatin and 5-fluorouracil in A549 lung adenocarcinoma cells by modulating oxidative stress, ER stress markers, and autophagy-related pathways *in vitro*. Co-treatment with OE and either chemotherapeutic agent significantly reduced cell viability and promoted autophagic activation without triggering classical apoptotic mechanisms.

The increase in LC3-II levels, the concomitant decrease in Beclin-1 expression, and the persistence of autophagic signaling despite 3-MA treatment suggest the activation of a non-canonical autophagic pathway. Importantly, ATG7 silencing experiments indicate that this process is Beclin-1–independent but requires ATG7, distinguishing it from fully alternative ATG7-independent autophagy and supporting the existence of a divergent regulatory mechanism.

**FIGURE 12:**
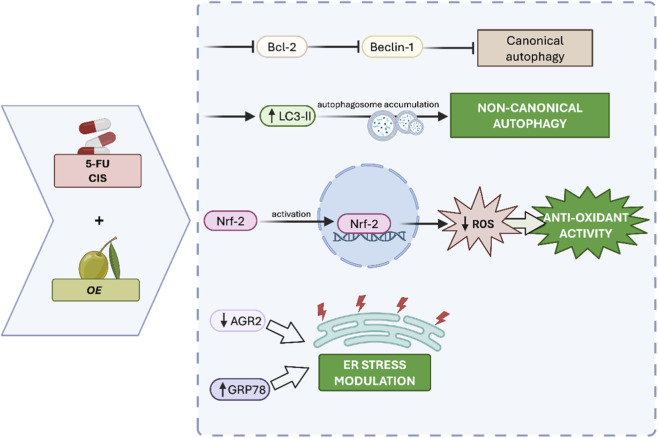
Proposed mechanism of action of OE in combination with 5-FU and CIS.

In parallel, OE reduced intracellular ROS levels and promoted Nrf2 activation (Pan et al., 2013), highlighting its antioxidant activity. Moreover, AGR2 downregulation and GRP78 upregulation further support a role for OE in modulating ER stress–related pathways (see Figure 12).

Although these findings underscore the potential of OE as a non-toxic adjuvant capable of enhancing chemotherapeutic efficacy through alternative cell death mechanisms, the results should be interpreted cautiously, as they derive from a single lung cancer cell line under *in vitro* conditions. Further studies using additional lung cancer models, advanced *ex vivo* systems, and *in vivo* approaches will be necessary to validate these observations and clarify the translational potential of OE in integrative oncology strategies.

## 5 Materials and methods

### 5.1 Cell cultures and compounds availability

The human lung adenocarcinoma cell line A549 was kindly provided by Prof. Rosalinda Sorrentino (Department of Pharmacy, University of Salerno). A549 cells were cultured at 37 °C in a humidified atmosphere containing 5% CO_2_ and 95% air in Dulbecco’s Modified Eagle Medium (DMEM; Gibco) supplemented with 10% heat-inactivated fetal bovine serum (FBS; Gibco), 1% L-glutamine, and 1% antibiotics (100 U/mL penicillin and 100 μg/mL streptomycin; Gibco).

Healthy immortalized human lung fibroblasts (hTERT-LF) were purchased from ATCC (Rockville, MD, United States) and maintained at 37 °C in a humidified atmosphere with 5% CO_2_ using Fibroblast Basal Medium fully supplemented according to the manufacturer’s instructions.

The olive fruit extract of *Olea europaea* L. (OE) used in this study was provided by Arterra Bioscienza S.r.l. (Naples, Italy) under a Material Transfer Agreement (MTA). The extract was originally supplied to Arterra Bioscienza by PhenoFarm S.r.l. (Naples, Italy) under the commercial name *Phenolea® Active Complex*, compliant with European Regulations No. 1829/2003 and No. 1830/2003. OE concentrations reported in the study refer to the final percentage (v/v) of the commercial extract preparation added to the culture medium.

Ergothioneine (ERGO), 5-fluorouracil (5-FU), and cisplatin (CIS) were purchased from Sigma-Aldrich (ERGO, cat. no. E7521; 5-FU, cat. no. 343922; CIS, cat. no. O9512) and prepared according to the manufacturer’s instructions.

### 5.2 Cell viability assays

To test the cytotoxicity of the molecules, hTERT-LF cells were cultured in 96-multiwell plates 24 h before the treatment at low-density of 1,500 cells/well and treated with OE at concentrations ranging from 1% to 5%; while as a positive control has been used ERGO at the concentration of 300 μM for 24 h. To test the effect of OE and the synergistic effect of OE with 5-FU and OE with CIS, A549 cells were cultured in 96- plates 24 h before the treatment at low-density of 1,500 cells/well and treated with 5% of OE, with 5-FU and CIS at concentrations ranging from 10 to 40 μM for 24 and 48 h. Each well contained 100 µL complete medium and was incubated at 37 °C, 5% CO_2_ for 24 h to allow cell adherence and growth. The medium was then replaced with a new complete medium containing OE, the combination of 5-FU with OE, and CIS with OE for 24 and 48 h. Cell viability was assessed by MTT assay at a concentration of 5 mg/mL. The cells were left in the incubator for 3 hours; at the end of the 3 hours the medium was aspirated and 70 μL of DMSO were added and the cells were left in the incubator for 15 min, the DMSO dissolved the Formezan crystals, and the plate was colored purple; the absorbance was measured at 570 nm using Perkin Elmer Victor X3 2030-0030 Multimode Microplate Reader. All experiments were performed in triplicate. The effect of molecules on cell viability was assessed as the percentage of viable cells compared to not-treated control cells, which were arbitrarily assigned a viability of 100%.

### 5.3 Calculation of synergic activity

To assess the interaction between chemotherapy and OE on cell viability, the Bliss Independence model was applied. This model assumes that the two agents act independently and estimates the expected combined effect based on their individual activities.

First, the viability of cells treated with each compound alone and in combination was measured and normalized to the untreated control (set at 100%). The effect of each treatment was then calculated as:

$$E=1‐\left({\text{Viability}}_{\text{treated}}\right)/\left({\text{Viability}}_{\text{control}}\right)$$

where E represents the fractional inhibition or effect, and viability values are expressed as a proportion (e.g., 70% viability = 0.7). The expected combination effect under the Bliss Independence model was calculated using the formula:

$$E_{\text{Bliss}}=E_{A}+E_{B}‐\left(E_{A}\cdot E_{B}\right)$$

where E_A_ and E_B_ are the effects of compounds A and B alone, respectively.

The observed combination effect (E_AB_) was also calculated using the formula above, replacing the measured viability of the combination.

Comparison of the observed effect (E_AB_) with the Bliss expected value (E_Bliss_) allowed classification of the interaction as follows:

- Synergistic: E_AB_ > E_Bliss_
- Additive: E_AB_ = E_Bliss_
- Antagonistic: E_AB_ < E_Bliss_

All values were calculated using Microsoft Excel (see Supplementary S1).

### 5.4 Preparation of total protein extracts from A549 cells

A549 cells were seeded in 6-well plates (8 × 10^5^ cells/well) and treated with OE at a concentration of 5% or in combination with 10 and 40 μM 5-FU or CIS for 24 h, and compared to not treated cells (only medium) or treated only with 10 and 40 μM 5-FU or only with 10 and 40 μM CIS. The same experimental procedure was applied to inhibit autophagy; however, after 24 h of treatment, 3-Methyladenine (3-MA) (Sigma-Aldrich, cat. no 189490) was added at a concentration of 2.5 mM 4 h. For ATG7 knockdown experiments, A549 cells were seeded at the same density (8 × 10^5 cells/well) and transfected with 50 nM siRNA targeting ATG7 (siATG7, Qiagen) or a non-targeting scramble control using Lipofectamine 3000 (Thermo Fisher Scientific) according to the manufacturer’s protocol. After 24 h of transfection, cells were treated with OE, 5-FU, CIS, or their combinations as described above. To obtain total protein extracts, cells were washed with cold PBS and resuspended in RIPA lysis buffer (1X) containing 50 mM Tris-HCl (pH 7.6), 150 mM NaCl, 5 mM EDTA, 0.5% NP-40, 0.5% sodium deoxycholate, 10% SDS, phosphatase, and Protease/Phosphatase Inhibitor Cocktail (Cell Signaling). Cell homogenates were centrifuged at 15,000 *g* for 30 min at 4 °C, and the supernatant was used as a total protein extract. The protein content of the total extracts was determined using the Bio-Rad protein assay reagent with bovine serum albumin as the standard.

### 5.5 Western blot analysis

Total protein extracts were analyzed on 12% SDS/polyacrylamide using 4X SDS Sample Loading Buffer. Following electrophoresis proteins were transferred onto a PVDF (Millipore) membrane (Bio-Rad Trans-Blot Apparatus) and probed with the rabbit polyclonal antibodies: anti-LC3A/B, anti-Bcl2, anti-caspase3, anti-cleaved caspase3, anti-Beclin-1 (all from Cell Signaling) and anti-ATG7 (abcam ab133528), diluted 1:1,000 in non-fat dried milk 5% in TTBS, and as an internal control the rabbit anti-β-actin monoclonal antibody (Cell Signaling) diluted 1:1,000 non-fat dried milk 5% in TTBS. All primary antibodies were incubated at 4 °C overnight. The appropriate secondary anti-rabbit HRP-conjugated antibody (Biorad) diluted 1:3000 in milk 5%, was added at room temperature for 1 h and immune-reactive proteins were detected using the ECL (Bio-Rad, United States, cod.1705061) according to the manufacturer’s instructions. Immunopositive bands were analysed by densitometry using the ImageJ software. The effect of treatment on protein expression was assessed as fold induction compared to not-treated control cells, which were arbitrarily assigned protein expression value equal to 1. All experiments were performed in duplicate.

### 5.6 RNA extraction and cDNA synthesis

Total RNA was extracted from A549 and hTERT-LF cells by mechanical homogenisation in Trizol Reagent according to the manufacturer’s protocol (Life Technologies). The amount of total extracted RNA was estimated by measuring the absorbance at 260 nm and the purity by 260/280 and 260/230 nm ratios by Nanodrop (ND-1000 UV–Vis Spectrophotometer; NanoDrop Technologies). RNA integrity was evaluated by agarose gel electrophoresis. For each sample, 1,000 ng of total RNA were retro-transcribed with iScriptTM cDNA synthesis kit (Bio-Rad), following the manufacturer’s instructions.

### 5.7 Gene expression by real-time qPCR

For real-time qPCR experiments, the data from each cDNA sample were normalized using the human housekeeping gene RLP0 (ribosomal protein lateral stalk subunit P0). The specific primers used for amplification of RLP0, AGR2 and GRP78 were designed based on the nucleotide sequences downloaded by UCSC Genome browser database using Primer3WEB v.4.0.0 (Table 2). A final concentration of 0.3 pmol/μL for each primer, 5 μL of 1:10 diluted cDNA having initial concentration of 1 μg and iTaq Universal SYBR Green Supermix (total volume of 20 μL) were used for the reaction mixture. PCR amplifications were performed in Bio-Rad CFX Opus 96 Real-Time PCR System using the following thermal profile: 95 °C for 10 min, one cycle for cDNA denaturation; 95 °C for 15 s and 60 °C for 1 min, 40 cycles for amplification; 72 °C for 5 min, one cycle for final elongation; and one cycle for melting curve analysis (from 60 °C to 95 °C) to verify the presence of a single product. Each assay included a negative control missing the cDNA template for each primers pair. Specificity of amplification reactions was verified by melting curve analysis. Calculations of relative expression levels were performed using the 2^−ΔΔCt^ method (Tang et al., 2014). All analysis was performed in triplicate to guarantee the accuracy of results.

**TABLE 2:** Primers used for amplification of RLP0, AGR2 and GRP78.

GENE	Fw	Rv
RLP0	5′-TGG CAG CAT CTA CAA CCC TG-3′	5′-GAC AAG GCC AGG ACT CGT TT-3′
AGR2	5′-ATG GAG AAA ATT CCA GTG TC-3′	5′-TTA CAA TTC AGT CTT CAG CA-3′
GRP78	5′-TTG GAG GTG GGC AAA-3′	5′-CCA GCA ATA GTT CCA-3′

### 5.8 ROS assay

A549 cells were seeded in 96-well plates (20 × 10^3^ cells/well) and treated with OE at a concentration of 5% or in combination with 10 and 40 μM 5-FU or CIS for 24 h and compared to not treated cells (only medium) or treated only with 10 and 40 μM 5-FU or only with 10 and 40 μM CIS. After the treatment, cells were washed twice with PBS and then the DCF at 25 μM was added. The reactions were incubated at 37 °C for 40 min. Fluorescence was measured using Multimode Microplate Reader, BioTek Synergy H1 (Agilent) (excitation wavelength 485 nm, emission wavelength 532 nm). All experiments were performed in triplicate. The fluorescence peak was used to represent the level of ROS production. The values were expressed as percentage (%) relative fluorescence as compared to not treated.

### 5.9 Immunofluorescence assay

For immunocytofluorescence, A549 cells were cultured at the density of 1 × 10^5^/cm^2^ in a eight well chamber-slide (Ibidi®) and fixed directly by 4% paraformaldehyde (PFA) for 15 min RT. Subsequently, cells were washed 3 times in 1X PBS. We removed the PBS and washed cells twice with freshly prepared IF Wash Buffer (1X PBS, 0.1% Tween ® 20). Samples were subsequently treated with blocking mix, IF Perm Buffer (1X PBS, 1% BSA, 0.1% Triton X-100), for 40 min at room temperature. Then, incubation with primary antibody was performed in blocking mix, IF Staining Buffer (1X PBS, 1% BSA), overnight at 4 °C. The day after, samples were washed in 1X PBS, 0.1% Tween ® 20, 5 times and incubated with a secondary antibody (1:1,000) in IF Staining Buffer, for 2 h at RT. Samples were finally washed in 1X PBS, 0.1% Tween ® 20 for 10 min, 3 times, counterstained with DAPI 1:1,000 (4′, 6′-diamidino-2-phenylindole).After that the cells were left in PBS1x in the chamber slides at 4 °C before acquisition. The following primary antibodies were used: Anti-Nrf2 protein 1:50. Secondary antibodies were conjugated of Alexa Fluor 488.

### 5.10 Images acquisitions and fluorescence analysis

Immunoprofiled cultured cells were photographed on an Evident FV400 confocal microscope, equipped with 20X, 40X and 63X objectives. All images were processed using Inkscape, GIMP2.6 software and ImageJ Cell counter plug-in.

For each experimental condition, five images were captured and analyzed using ImageJ software. A region of interest (ROI) was selected in each image, expressed in pixel^2^ (or in calibrated units), and the Mean was subsequently calculated. The Mean refers to the average pixel intensity (grayscale value) within the selected ROI. Specifically, it corresponds to the arithmetic mean of all pixel values inside the ROI. In the case of color images converted to grayscale, the Mean reflects the average brightness intensity. Data are expressed as ratios, normalized to the mean intensity value observed in the untreated control sample (set as 1).

### 5.11 Statistical analysis

All statistical analyses were performed using GraphPad Prism 8.0.1 (GraphPad Software Inc., La Jolla, CA, United States). All experiments were performed with three independent biological replicates, each with two technical replicates. Data were expressed as the means ± standard deviations. The Student’s t-test or analysis of variance One-way ANOVA followed by Dunnett’s multiple comparisons test with values of p < 0.05 considered significant (Wirawan et al., 2012).

## Data Availability

All data presented in the study are included in the article/Supplementary Material, further inquiries can be directed to the corresponding author/s.

## Supplementary material

Supplementary Figure 1 Western blot analysis after siRNA ATG7 transfection. (A) Western blot analysis of ATFG7, LC3A/B-I, LC3A/B-II, and Beclin-1 expression in A549 cells transfected with 50nM of siATG7 and after treated for 24 h with indicated doses of OE, 5-FU, CIS, and combined treatments. Bands represent one out of three separate experiments performed. (B) Densitometric analysis. For ATG7 and LC3A/B Western blot analysis, equal protein loading and transfer efficiency were verified by Ponceau S staining of the membrane. Data are expressed as mean ± SD (standard deviation), n = 3. For ATG7, the significance was determined by the student’s t-test (p < 0.05), representing significance compared to not treated (NT). For LC3A/B, the significance was determined by One-way ANOVA followed by Dunnett’s multiple comparisons test (p < 0.05), (p < 0.01), * (p < 0.001), represents significance LC3A/B-II compared to not treated (NT); # (p < 0.05), ## (p < 0.01), ### (p < 0.001), represents significance LC3A/B-II compared to Scramble.

## Glossary

LC: lung cancer
OE: *Olea europaea L.*
A549: human lung epithelial adenocarcinoma cells
NSCLC: non-small-cell lung cancer
CIS: Cisplatin
5-FU: 5-fluorouracil
LC3A/B: Microtubule-associated protein 1A/1B-light chain 3
AGR2: anterior gradient 2
GRP78: glucose-regulated protein 78
RPLP0: ribosomal Protein Lateral Stalk Subunit P0
ERGO: ergothioneine
GGT: γ‐glutamyl transpeptidase
NT: untreated
hTERT-LF: human telomerase reverse transcriptase lung fibroblast
ROS: reactive oxygen species
IFN-γ: interferon gamma
NRF2: nuclear factor erythroid 2-related factor 2
ER: endoplasmatic reticulum
NF-κB: nuclear factor kappa-light-chain-enhancer of activated B cells
3-MA: 3-methyladenine
DCF: diclorofluorescein
DMSO: dimethyl Sulfoxide
RIPA: radioimmunoprecipitation assay buffer
RT-qPCR: quantitative reverse transcription polymerase chain reaction
EDTA: ethylenediaminetetraacetic acid
PBS: phosphate-buffered saline
BSA: bovine serum albumin
SDS: sodium dodecyl sulfate
DAPI: 4′, 6′-diamidino-2-phenylindole
IF: immunofluorescence
RNA: ribonucleic Acid
cDNA: complementary deoxyribonucleic acid
