# Supplementary figures and images for: Enhancement of chemotherapeutic efficacy via non-canonical autophagy induced by Olea europaea in human model of lung adenocarcinoma cells (A549)
Source: Front Cell Dev Biol. 2026 Apr 24;14:1760977. doi: 10.3389/fcell.2026.1760977 (PMC13153436; doi:10.3389/fcell.2026.1760977)

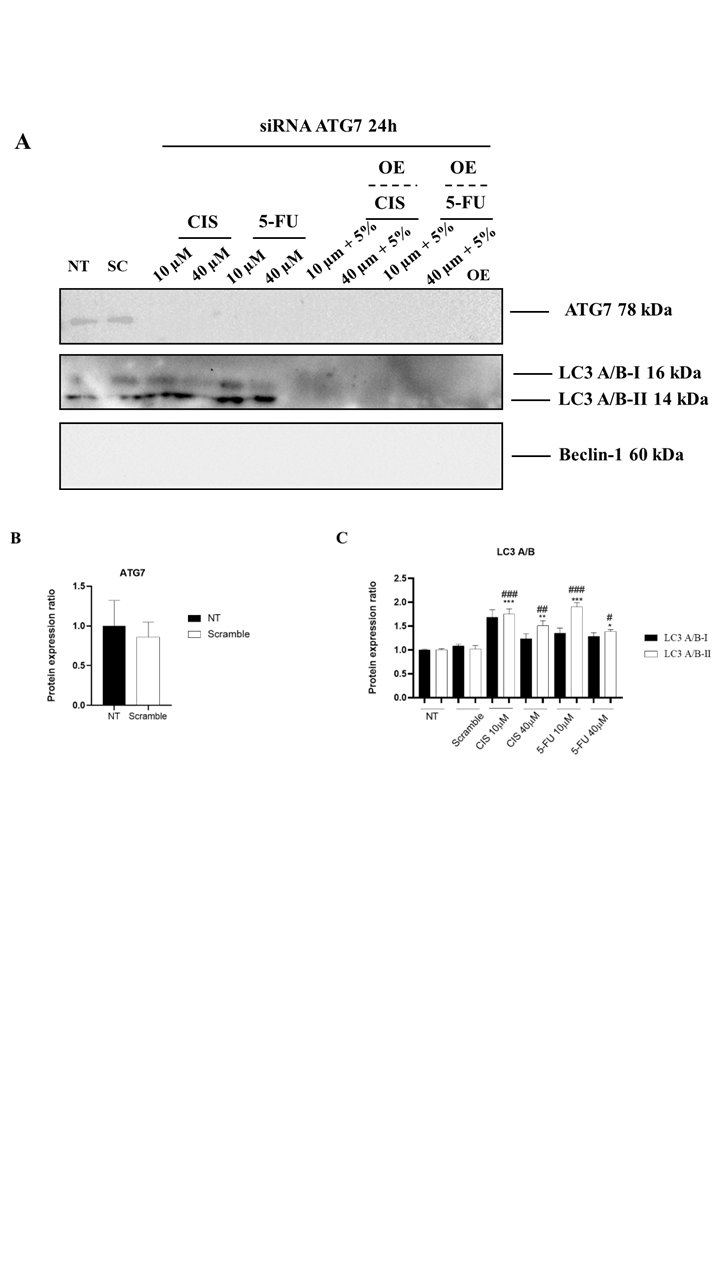

Supplement: Supplementary file 1 [file Image1.tif]
